# Supplementary material for: Functional Analysis of Pheromone Biosynthesis Activating Neuropeptide Receptor Isoforms in Maruca vitrata
Source: Cells. 2023 May 17;12(10):1410. doi: 10.3390/cells12101410 (PMC10217269; doi:10.3390/cells12101410)
Supplement: Supplementary file 1 [file cells-12-01410-s001.zip › Figure S1.pdf]

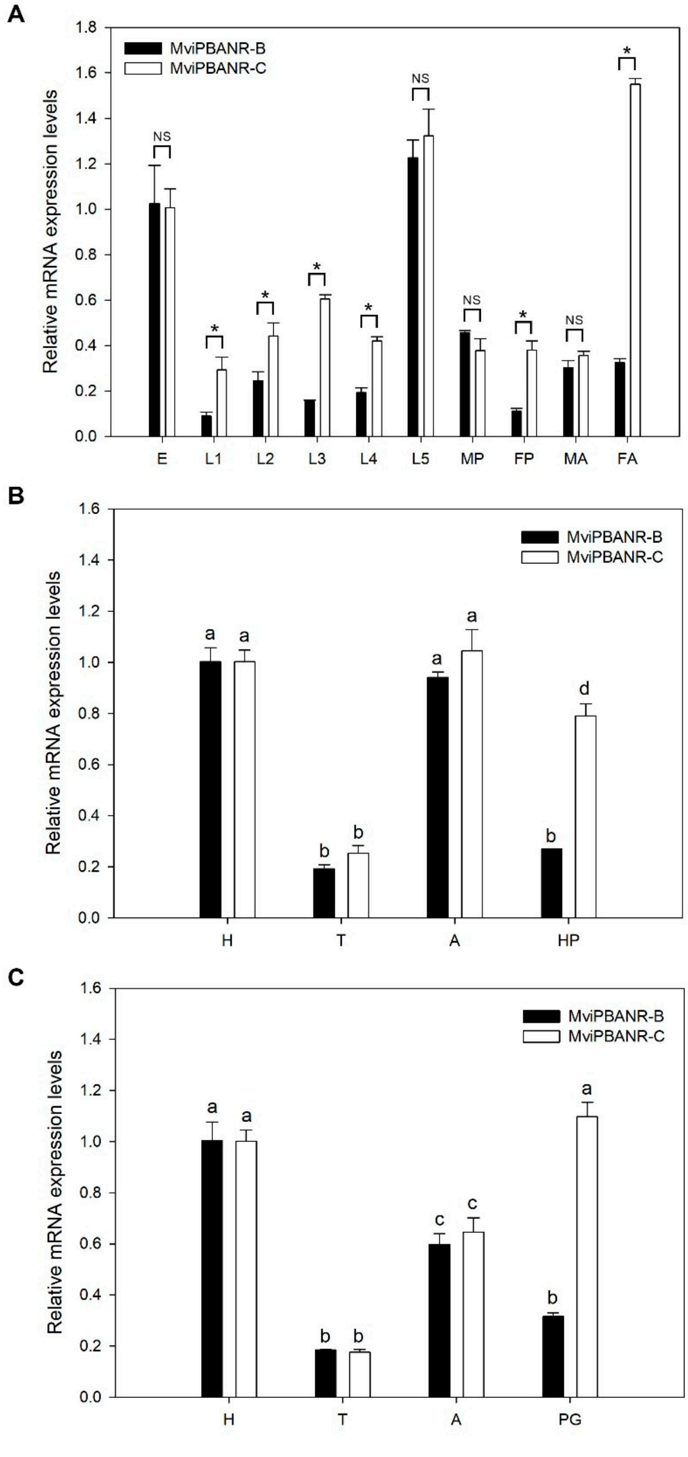

Figure S1. *In vivo* gene expression of MviPBANR-B and MviPBANR-C. (**A**) Developmental stage-specific expression. Egg, different instar larvae (L1-L5), pupa (P), and adult (A) were analyzed. For pupae and adults, male (M) and female (F) individuals were analyzed. The statistical differences were analyzed by *t*-test (*P <

0.001; NS, no significance). (**B**) Tissue-specific expression in male adults; head (H), thorax (T), abdomen (A), and hair-pencil (HP) were analyzed. The statistical differences were analyzed by ANOVA (P < 0.001). (**C**) Tissue-specific expression in female adults; head (H), thorax (T), abdomen (A), and pheromone gland (PG) were analyzed. RPS18 was used as a positive control for the qRT-PCR. Different letters above each bar indicate a statistical difference by ANOVA (P < 0.001).
