# Supplementary material for: Functional Analysis of Pheromone Biosynthesis Activating Neuropeptide Receptor Isoforms in Maruca vitrata
Source: Cells. 2023 May 17;12(10):1410. doi: 10.3390/cells12101410 (PMC10217269; doi:10.3390/cells12101410)
Supplement: Supplementary file 1 [file cells-12-01410-s001.zip › Figure S2.pdf]

A

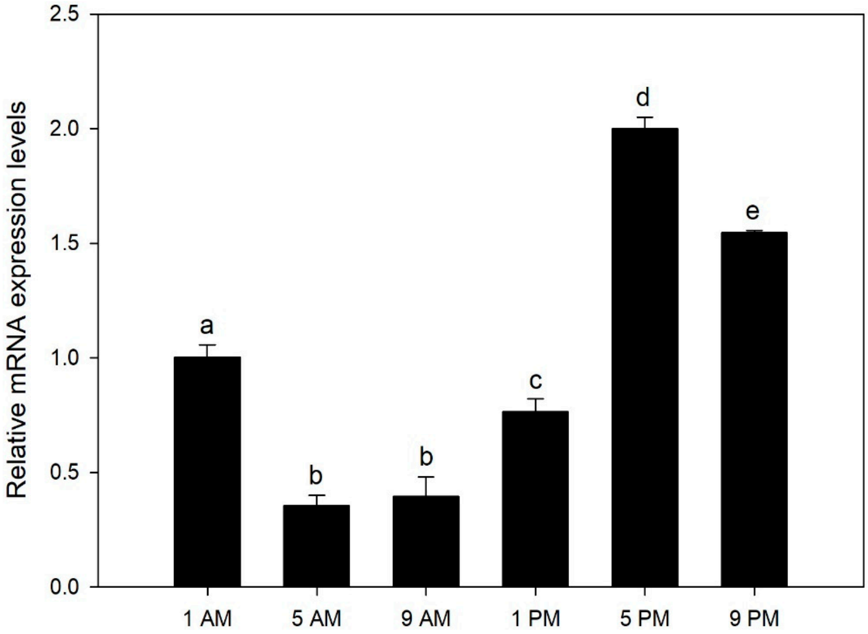

B

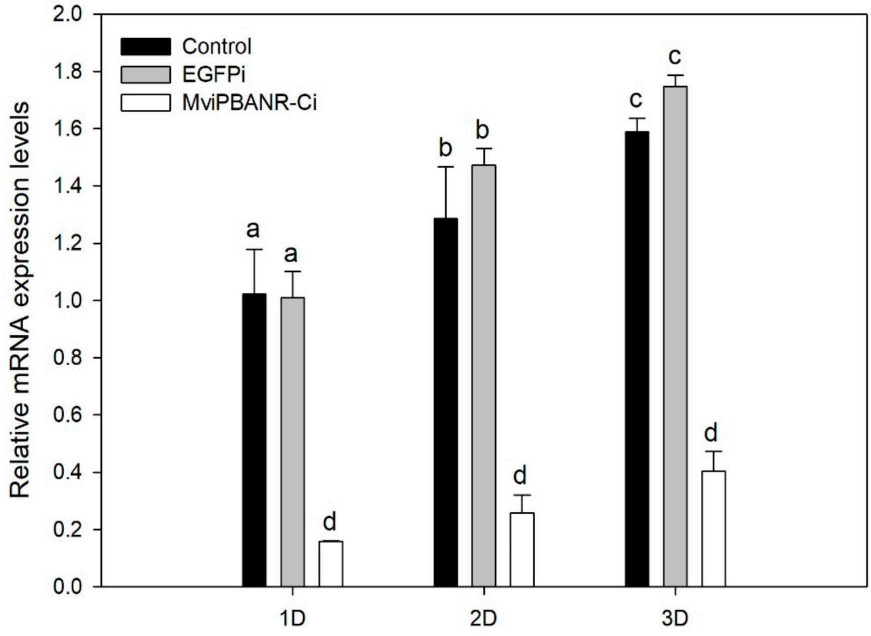

Figure S2. Expression of MviPBANR-C after RNAi in female adults of *M. vitrata*. (**A**) Diurnal expression of MviPBANR-C in pheromone glands. (**B**) Suppression of MviPBANR-C after RNAi in the pheromone glands. EGPF-specific dsRNA was injected as a negative control. The expression levels of MviPBANR-C against RPS18 were measured by qRT-PCR. Different letters above each bar indicate a statistical difference by ANOVA ($P < 0.001$).
