# Supplementary material for: Functional Analysis of Pheromone Biosynthesis Activating Neuropeptide Receptor Isoforms in Maruca vitrata
Source: Cells. 2023 May 17;12(10):1410. doi: 10.3390/cells12101410 (PMC10217269; doi:10.3390/cells12101410)
Supplement: Supplementary file 1 [file cells-12-01410-s001.zip › Table S1.pdf]

Table S1. Lists of primer sequences used in this study

Name	Sequence (5'-3')	Purpose
MvPBANR-B-FP	ATGGAGATTAACTCGTCGGAACTACAGATGC	Cloning of MviPBANR isoforms
MvPBANR-B-RP	TCACGTGACGCCGCCTATGTTG	
MvPBANR-C-FP	ATGGAGATTAACTCGTCGGAACTAC	
MvPBANR-C-FP	TCACGGCGGGCAGATCGG	
RT-MvPBANR-B-FP	AGCCTCAACAGCCCCGATCTG	1) Amplification of MviPBANR isoforms for qPCR<br>2) conformation of transfection with MviPBANR isoforms in HeLa cells
RT-MvPBANR-B-RP	CGTGACGCCGCCTATGTTGCAG	
RT-MvPBANR-C-FP	TGCGGGCACAGGAGCGGG	
RT-MvPBANR-C-FP	CGGGCAGATCGGGGCCGTTGAG	
Ex-MvPBANR-B-FP	GGTACCGCAATGGAGATTAACTCG	Amplification of MviPBANR isoforms for expression vector construction
Ex-MvPBANR-B-RP	GAATTCTCACGTGACGCCGCCTATG	
Ex-MvPBANR-C-FP	GGTACCGCAATGGAGATTAACTCGTCGGAACTAC	
Ex-MvPBANR-C-RP	GAATTCTCACGGCGGGCAGATCGG	
T7-MvPBANR-C-FP	TAATACGACTCACTATAGGGAGATGCGGGCACAGGAG CGGG	Amplification of MviPBANR-C and EGFP for dsRNA synthesis
T7-MvPBANR-C-RP	TAATACGACTCACTATAGGGAGACGGGCAGATCGGGG CCGTTGAG	
T7-EGFP-FP	TAATACGACTCACTATAGGGAGACACCTTGATGCCGTT CTTCT	
T7-EGFP-RP	TAATACGACTCACTATAGGGAGAGCTGACCCTGAAGTT CATCTG	
EF1A-FP	GAACGTGTCGGTGAAAGAGC	Amplification of EF1-α and RPS18 as positive controls
EF1A-RP	CGCGTCACCGGATTTTATGG	
RPS18-FP	GGACGTAGATATTCCAACATTGTG	
RPS18-RP	GCTGTATTTACCGTCAACAATGTC	
GAPDH-FP	GAAGGTGAAGGTCGGAGTC	Amplification of GAPDH as a positive control
GAPDH-RP	GAAGATGGTGATGGGATTTC	
